# Numerical Simulation and Comparison of Conventional and Sloped Solar Chimney Power Plants: The Case for Lanzhou

**DOI:** 10.1155/2013/852864

**Published:** 2013-12-30

**Authors:** Fei Cao, Huashan Li, Yang Zhang, Liang Zhao

**Affiliations:** ^1^College of Mechanical and Energy Engineering, Jimei University, Xiamen 361021, China; ^2^State Key Laboratory of Multiphase Flow in Power Engineering, Xi'an Jiaotong University, Xi'an 710049, China; ^3^CAS Key Laboratory of Renewable Energy, Guangzhou Institute of Energy Conversion, Chinese Academy of Sciences, Guangzhou 510640, China; ^4^University of Chinese Academy of Sciences, Beijing 100049, China; ^5^Nuclear and Radiation Safety Center, Beijing 100082, China

## Abstract

The solar chimney power plant (SCPP) generates updraft wind through the green house effect. In this paper, the performances of two SCPP styles, that is, the conventional solar chimney power plant (CSCPP) and the sloped solar chimney power plant (SSCPP), are compared through a numerical simulation. A simplified Computational Fluid Dynamics (CFD) model is built to predict the performances of the SCPP. The model is validated through a comparison with the reported results from the Manzanares prototype. The annual performances of the CSCPP and the SSCPP are compared by taking Lanzhou as a case study. Numerical results indicate that the SSCPP holds a higher efficiency and generates smoother power than those of the CSCPP, and the effective pressure in the SSCPP is relevant to both the chimney and the collector heights.

## 1. Introduction

**Figure 1:**
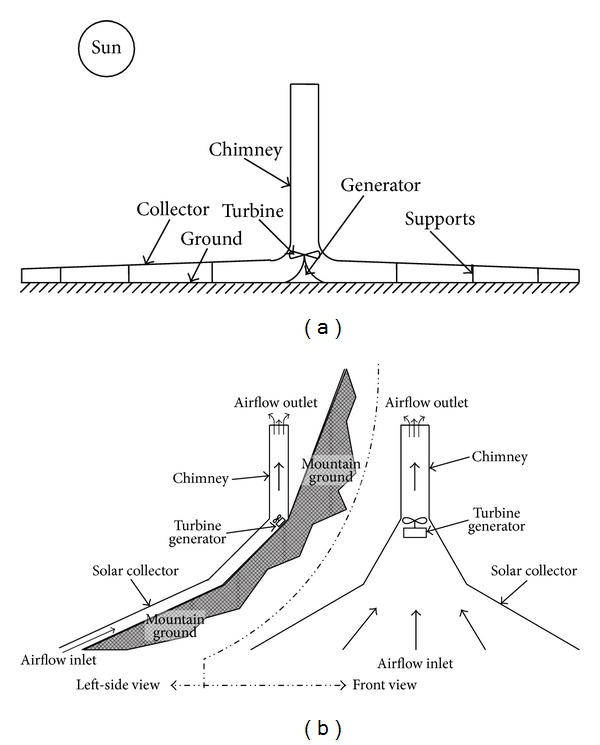
Schematic of the solar chimney power system: (a) on horizontal surface, (b) on sloped surface.

The solar chimney power plant (SCPP) offers interesting opportunities to use the clean solar radiation to satisfy the ever-increasing world energy demand. It is designed to produce electric power on a large-scale by first converting solar energy into thermal energy which is then converted into kinetic energy to drive the wind turbine for power generation. There are many styles of SCPPs reported in the literature, among which the conventional solar chimney power plant (CSCPP) and the sloped solar chimney power plant (SSCPP) are most suitable for Northwest China [1]. The schematics of the CSCPP and the SSCPP are shown in Figure 1.

The solar chimney power plant concept was originally proposed in 1903 by Isidoro Cabanyes [2]. In 1931, a description of a solar chimney power plant was presented by Günther [3]. The basic study on the solar chimney concept was performed by Schlaich in the 1970s, and in 1981 he began the construction of a 50 kW pilot solar chimney power plant in Manzanares, Spain [4, 5]. The chimney tower is 194.6 m in height, and the collector radius is 122 m. Many studies on the CSCPP have been presented in the literature on its analytical and experimental performances [6–9]. However, the high chimney reduces the feasibility of the SCPP because of its safety risks. In 2005, a sloped solar chimney power plant (SSCPP) was proposed by Bilgen and Rheault [10], whose solar collector is laid along the hillside. Further studies indicated that the SSCPP held higher thermal efficiency and could somehow reduce the chimney height [1, 11].

Numerical simulations of the CSCPP have been well documented in the literature. Ming et al. employed Computational Fluid Dynamics (CFD) software to evaluate the performance of a CSCPP, in which the effects of various parameters on the relative static pressure, the driving force, the power output, and the efficiency were further investigated with or without a turbine [12, 13]. Pastohr et al. simulated the temperature and velocity fields in the CSCPP using numerical solutions [14]. Dimensionless analyses of CSCPPs were carried out, using the finite volume method, to understand the flow through the solar collector and chimney [15]. Zhou et al. built a mathematical model to simulate the compressible flow through the solar chimney [16]. These studies make clear the flow details in the CSCPP, through which the structures of the CSCPP are optimized. However, the numerical simulations on the SSCPP have rarely been reported. The case studies carried out by Cao et al. [1, 11] and Bilgen and Rheault [10] only estimated the monthly average performance of the SSCPPs. No further details of the flow, temperature, and pressure fields can be obtained in the literature. Considering this, a numerical simulation is carried out to analyze and compare the monthly average performance and inner details of the CSCPP and the SSCPP by taking Lanzhou, China, as a case study.

## 2. Mathematical Model

### 2.1. Basic Equations

The continuity equation, the 3D Navier-Stokes equations, the energy equation, and the *k*-*ε* equations are described as follows.

Continuity:

(1) $$\begin{array}{c} \frac{\partial \rho }{\partial t}+\frac{\partial }{\partial x_{i}}\left(\rho u_{i}\right)=0. \end{array}$$

Navier-Stokes equations:

(2) $$\begin{array}{c} \frac{\partial }{\partial t}\left(\rho u_{i}\right)+\frac{\partial }{\partial x_{j}}\left(\rho u_{i}u_{j}\right)=-\frac{\partial p}{\partial x_{i}}+\frac{\partial \tau_{ij}}{\partial x_{j}}+\rho g_{i}. \end{array}$$

Energy equation:

(3) ∂∂t(ρcpT)+∂∂xj(ρcpujT) =∂∂xj(k∂T∂xj)+τij∂ui∂xj+βT(∂p∂t+uj∂p∂xj).

*k*-*ε* equations:

(4) ρDkDt=∂∂xi[(μ+μtσk)∂k∂xi]+Gk+βgiμtPrt∂T∂xi−ρε,ρDεDt=∂∂xi[(μ+μtσε)∂ε∂xi] +C1εεk(Gk+C3εβgiμtPrt∂T∂xi)−C2ερε2k,

where *ρ* is the airflow density, *t* is the time, *u* denotes the airflow velocity at the three directions (namely, the *i*, *j*, and *k* directions) in a Cartesian coordinate, *c*_*p*_ is the airflow specific heat capacity, *p* is the pressure, *β* is the thermal expansion coefficient, *T* is the airflow temperature, and *G*_*k*_ represents the generation of turbulence kinetic energy.

### 2.2. Solar Collector

**Figure 2:**
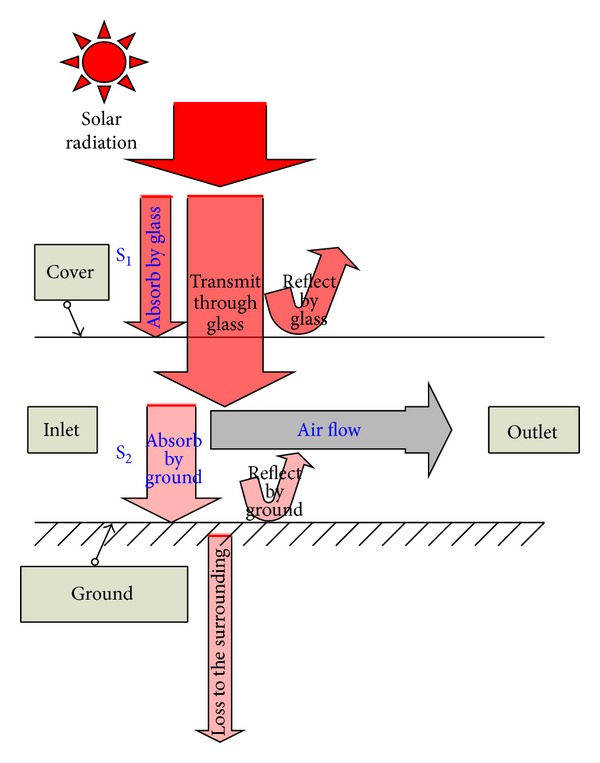
Thermal balance in the solar collector.

The solar collector of an SCPP can be basically treated as a solar air heater. The schematic of thermal balance in the collector is shown in Figure 2. The sunlight is transmited through the collector glass cover and it heats the ground below. The hot ground transfers heat in the style of convection and radiation to the air above it. The solar radiation absorbed by the glass cover is S_1_ and the solar radiation transferred through the glass cover and absorbed by the ground is S_2_. The cold air enters the collector and is heated by the hot ground.

The ray tracing algorithm of the solar load model is used to calculate the illumination energy source of the solar collector and ground, which results from the incident solar radiation. The heat flux produced is then coupled with the ANSYS Fluent calculation via a source term in the energy equation. The heat sources are added directly to the computational cells bordering each face and are assigned to adjacent cells. As the steady state, the average sun position vector and illumination parameters are set monthly. As a turbulence model is necessary for the description of the turbulent flow conditions, the standard model and standard wall mode are selected to describe the fluid flow inside the collector and the chimney.

### 2.3. Power Generation and System Efficiency

The density of the hot air in the chimney is smaller than the ambient air, resulting in a low static pressure in the chimney. The pressure difference between the chimney base and the ambient, Δ*p*_*t*_, is the system driving force to impel air to flow through the SCPP. The power generated by the turbine, *P*_ele_, and the overall efficiency, *η*, are, respectively,

(5) $$\begin{array}{c} P_{\text{ele}}=\eta_{t}\Delta p_{t}V_{\text{chi}}A_{\text{chi}}, \end{array}$$

(6) $$\begin{array}{c} \eta =\frac{P_{\text{ele}}}{\left(I_{\text{ra}}A_{\text{gro}}\right)}, \end{array}$$

where *η*_*t*_ is the efficiency of the turbine, *V*_chi_ is the velocity of the air in the outlet of the chimney, *A*_chi_ is the area of the outlet of the chimney, *I*_ra_ is the incident radiation of the sun, and *A*_gro_ is the area of ground.

## 3. Results and Discussion

The numerical simulation based on (1)–(6) is developed on ANSYS Fluent to simulate the performances of a CSCPP and a SSCPP in Lanzhou, China. Lanzhou (103.50°E, 36.03°N) is a zonal basin city 1520 m above the sea level, with an area of 13085.6 km^2^. It is the capital of Gansu Province and locates in the geographical central of Northwest China. Its annual global solar radiation is more than 5020 MJ/m^2^, and sunshine duration is over 2600 hours per year. Its annually mean temperature is 9.8°C.

**Figure 3:**
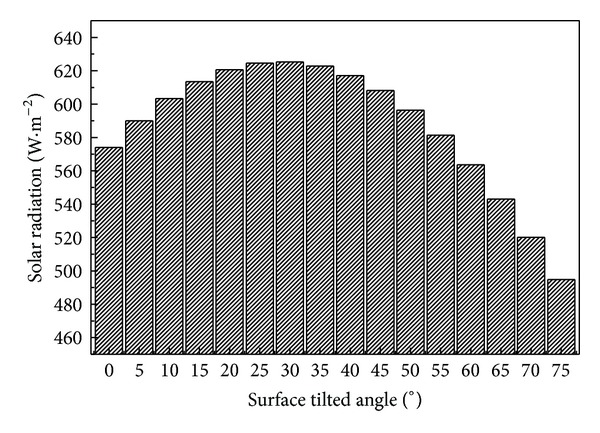
Annually average solar radiation for tilted surfaces.

As for the SSCPP, the solar collector tilted angle is an important parameter.  Figure 3 shows the annually average received solar radiation by the solar collectors at different surface angles. Near 30°, there is a pink point, where the solar collectors receive the maximum solar radiation (MSR). On the basis of the existing meteorological data, we choose 31°, which is named as the MSR angle, as the solar collector angel for the SSCPP.

The physical model for CSCPP is built based on the geometrical dimensions of the Manzanares prototype. To compare the performances between the SCPPs, the SSCPP has the same geometrical dimensions as the CSCPP. The main parameters used in the simulation process are summarized in Table 1.

The SCPP is divided into five areas, namely, the collector, the collector inlet, the chimney, the ground, and the chimney outlet. The assumed boundary conditions are illustrated in Table 2. The heat transfer coefficient between the glass roof and the ambient air, *h*_*r*_, is set as 4 W/(m^2^·K). The absorptivity and transmissivity of the glass are 0.1 and 0.8, respectively. The direct visible absorptivity and direct IR absorptivity of the ground surface are 0.8 and 0.92, respectively.

### 3.1. Model Validation

**Figure 4:**
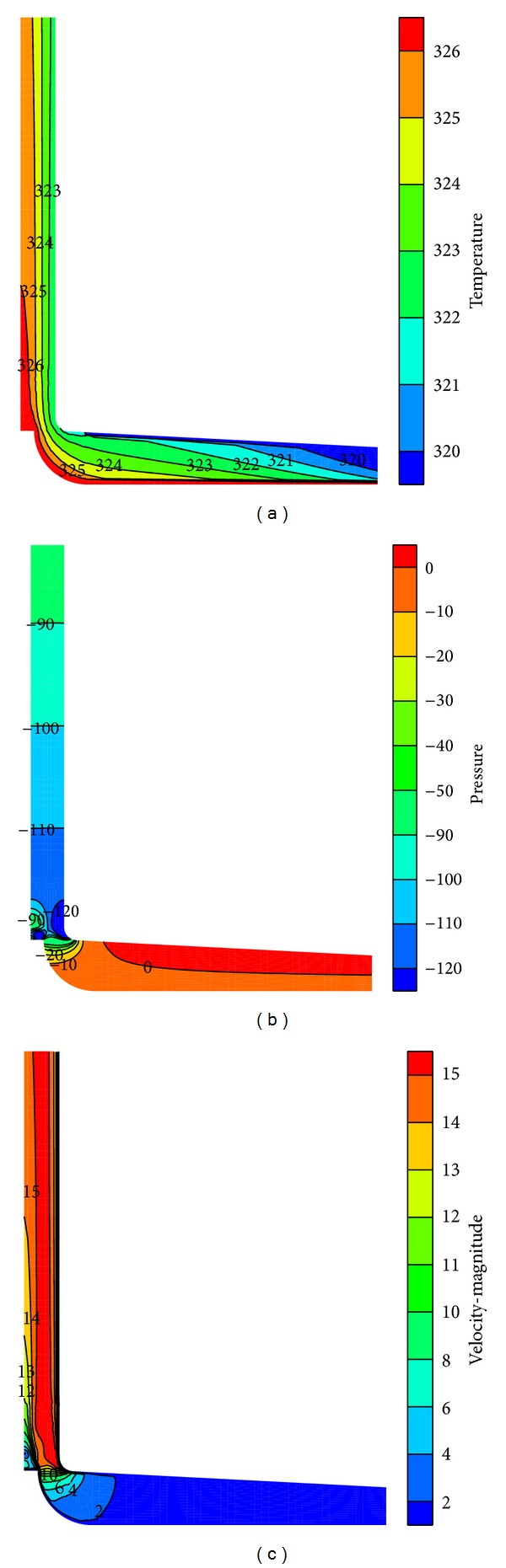
Temperature, velocity, and pressure fields of the CSCPP when *I*_ra_ = 1000 W/m^2^ (temperature unit: K, pressure unit: Pa, and velocity unit: m/s).

To validate the numerical model, the temperature rise in the collector and the upwind velocity at the chimney inlet are compared with the experimental data from the Spanish prototype [4]. The Spanish prototype experimental results indicate that, when the solar radiation is 1000 W/m^2^, the upwind velocity at the chimney base is 15 m/s and the temperature increase through the collector under noload conditions reaches 20 K. The temperature, velocity, and pressure fields of the simulated CSCPP are shown in Figure 4. And the comparison of the reported and simulated results is shown in Table 3. Through Figure 4 and Table 3, good quantitative agreements are observed between the experimental and the CFD results. Correspondingly, the built numerical model is ready to simulate the SCPP performance at other conditions.

### 3.2. Performance Comparison of**  **SCPPs

**Figure 5:**
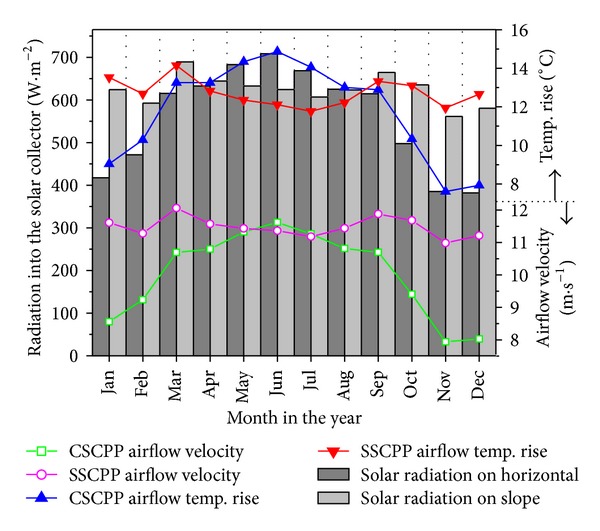
Monthly average energy input, temperature, and updraft flow velocities of the CSCPP and SSCPP.

The monthly average solar radiation on the horizontal and the slope, the CSCPP and SSCPP airflow temperature increase, and the updraft wind velocities are presented in Figure 5. It is observed from the figure that the SSCPP solar collector with MSR angle receives much higher radiation from January to March and from September to December than the CSCPP's does, leading to much higher energy absorption through the year (see Figure 3). Due to the different energy absorption, the temperature increases of the CSCPP and SSCPP also differ with each other. The solar radiation is found to be the major influence on the temperature, and the ambient temperature is the minor factor. The airflow velocities are observed to have similar tendency as the temperature increases for CSCPP and SSCPP, respectively. The tendencies of velocity and temperature fluctuation are similar to the solar radiation distribution in the year. The temperature rise and the airflow velocity of the SSCPP are much steadier than those of the CSCPP.

**Figure 6:**
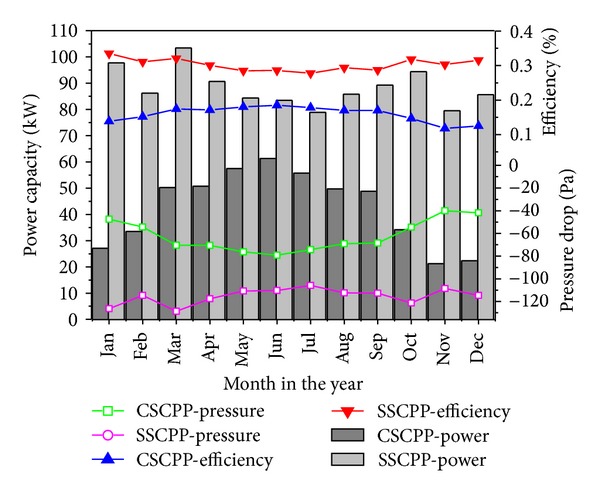
Pressure, efficiency, and power capacity of the CSCPP and SSCPP in each month.

The power, efficiency, and pressure of the CSCPP and the SSCPP are shown in Figure 6. The power and efficiency of the SSCPP are much higher than those of the CSCPP. Possible reason is that the SSCPP effective height is a result of the collector height and the chimney height. As for the SSCPP, its chimney height is the same as that of the CSCPP but it still holds the positive effect of the collector height, which we think generates a larger pressure difference and also contributes more to the updraft airflow.

### 3.3. CSCPPs and SSCPPs Inner Performance Comparison

**Figure 7:**
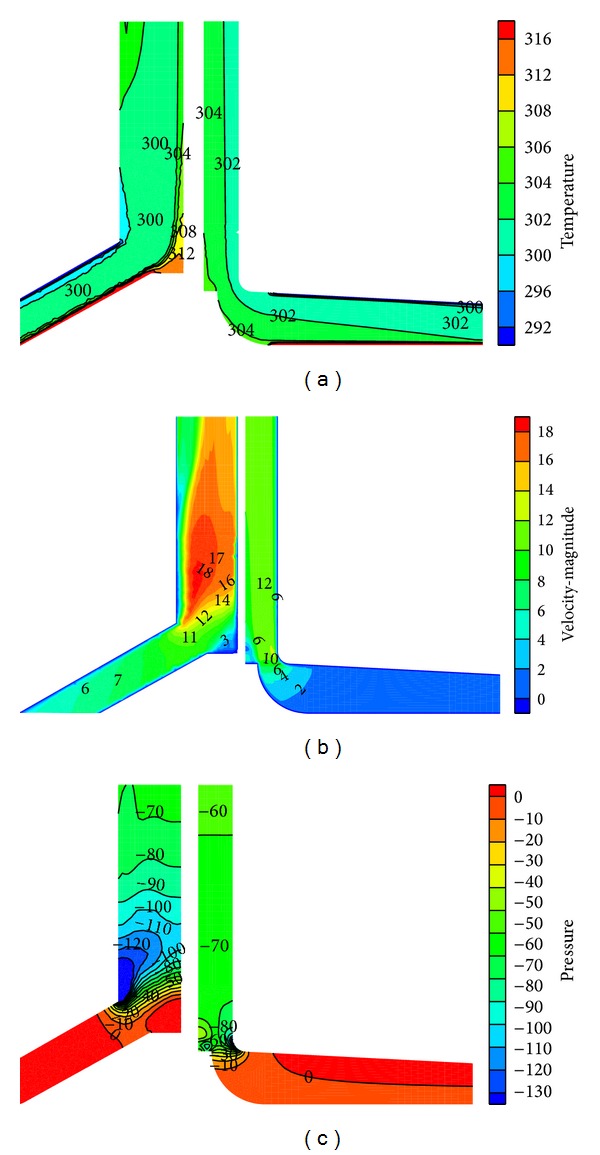
Comparison of the CSCPP and the SSCPP inner detail performance (temperature unit: K, velocity unit: m/s, and pressure unit: Pa).

Figure 7 shows the comparison of the temperature, pressure, and velocity distributions of the referred CSCPP and SSCPP in September. It is found from the figure that the solar radiation and airflow temperature of the CSCPP and the SSCPP are almost the same in September. Also, it can be observed that the velocity and pressure of the SSCPP are much higher than those of the CSCPP in the chimney at the same location. The temperature increase in the collectors of the CSCPP and SSCPP is similar because of the same input radiation (see Figure 5). An obvious eddy is found at the connection of the collector and chimney, where the turbine is practically constructed. Further optimization is needed for the SSCPP to weaken the airflow turbulence.

### 3.4. Effects of the Chimney Height on SSCPPs

**Figure 8:**
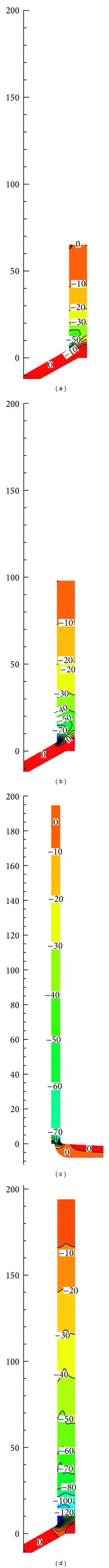
Inner pressure field comparison of four SCPPs with different chimney heights (pressure unit: Pa).

In order to make the effective height of the SSCPP clear, three SSCPPs whose chimney heights are 60 m, 98 m and 194.6 m, respectively, are compared under the same boundary conditions. A CSCPP with the chimney height being 194.6 is set as the reference. The pressure fields of the four SCPPs are presented in Figure 8. The collector and chimney heights of the four referred SCPPs are summarized in Table 4. It is found from Figure 8 that the SSCPP with the chimney height of 98 m holds similar pressure field in the chimney and similar effective pressure to the reference CSCPP. By analyzing Table 4, it is further observed that the SSCPP effective pressure is a function of (*H*_col_/2 + *H*_chi_), which agrees well with the analytical results reported by Bilgen and Rheault [10].

## 4. Conclusions

The inner detail performances of the CSCPP and SSCPP are compared through the numerical solution in this paper. Comparison analyses are also carried out to the SSCPPs with different chimney heights. Results indicate that the SSCPP holds much smoother performance than the CSCPP throughout the year. The effective pressure in the SSCPPs is a function of (*H*_col_/2 + *H*_chi_). An obverse airflow eddy is observed at the connection of the solar collector and the chimney in the SSCPP. Further optimization work is then suggested by the authors with the purpose of averaging the pressure distribution at the chimney section.

## Figures and Tables

**Table 1:** Main parameters of the SCPPs.

Parameter	Value
Collector square	46759.5 m^2^
Chimney height	194.6 m
Chimney radius	5.08 m
CSCPP collector radius	122 m
SSCPP collector height	193.5 m

**Table 2:** Boundary condition assumptions for the SCPPs in Fluent.

Location	Type	Description
Glass roof	Wall	Mixed, semitransparent
Ground surface	Wall	*q* = 0 W/m^2^, opaque
Chimney wall	Wall	*q* = 0 W/m^2^
Collector inlet	Pressure inlet	Δ*p* = 0 Pa
Chimney outlet	Pressure outlet	Δ*p* = 0 Pa

**Table 3:** The comparison of the numerical result and the experimental data.

Source	Temperature increase	Upwind velocity
Experimental data	20 K	15 m/s
Fluent result	21.5 K	14.33 m/s

**Table 4:** Collector and chimney heights of the four SCPPs.

No.	*H*_col_ (m)	*H*_chi_ (m)	*H*_col_/2 + *H*_chi_ (m)	Δ*P*_*t*_ (Pa)
c	0	194.6	194.6	68.5
a	193.5	60	156.75	42.8
b	193.5	98	194.75	62.1
d	193.5	194.6	261.35	112.7

## References

[1] Cao F, Zhao L, Li HS, Guo LJ (2013). Performance analysis of conventional and sloped solar chimney power plants in China. *Applied Thermal Engineering*.

[2] Lorenzo J Las Chimneas solares: de una propuesta espanola en 1903 a de Mansanares. http://www.fotovoltaica.com/chimenea.pdf.

[3] Günther H (1931). *Hundred Years-Future Energy Supply of the World*.

[4] Haaf W (1984). Solar chimneys, part II: preliminary test results from the Manzanares pilot plant. *International Journal of Solar Energy*.

[5] Haaf W, Friedrich K, Mayr G, Schlaich J (1983). Solar chimneys, part I: principle and construction of the pilot plant in Manzanares. *International Journal of Solar Energy*.

[6] Pasumarthi N, Sherif SA (1998). Experimental and theoretical performance of a demonstration solar chimney model-part I: mathematical model development. *International Journal of Energy Research*.

[7] Pasumarthi N, Sherif SA (1998). Experimental and theoretical performance of a demonstration solar chimney model, part II: experimental and theoretical results and economic analysis. *International Journal of Energy Research*.

[8] Pretorius JP, Kröger DG (2006). Critical evaluation of solar chimney power plant performance. *Solar Energy*.

[9] Zhou X, Yang J, Xiao B, Hou G (2007). Experimental study of temperature field in a solar chimney power setup. *Applied Thermal Engineering*.

[10] Bilgen E, Rheault J (2005). Solar chimney power plants for high latitudes. *Solar Energy*.

[11] Cao F, Zhao L, Guo L (2011). Simulation of a sloped solar chimney power plant in Lanzhou. *Energy Conversion and Management*.

[12] Ming T, Liu W, Xu G (2006). Analytical and numerical investigation of the solar chimney power plant systems. *International Journal of Energy Research*.

[13] Ming TZ, Liu W, Xu G (2008). Numerical simulation of the solar chimney power plant systems coupled with turbine. *Renewable Energy*.

[14] Pastohr H, Kornadt O, Gürlebeck K (2004). Numerical and analytical calculations of the temperature and flow field in the upwind power plant. *International Journal of Energy Research*.

[15] Chergui T, Larbi S, Bouhdjar A (2010). Thermo-hydrodynamic aspect analysis of flows in solar chimney power plants—a case study. *Renewable and Sustainable Energy Reviews*.

[16] Zhou X, Yang J, Xiao B, Hou G, Wu Y (2009). Numerical investigation of a compressible flow through a solar chimney. *Heat Transfer Engineering*.

